# The burden of non-communicable diseases and their related risk factors in the country of Georgia, 2015

**DOI:** 10.1186/s12889-019-6785-2

**Published:** 2019-05-10

**Authors:** Steven Russell, Lela Sturua, Chaoyang Li, Juliette Morgan, Marina Topuridze, Curtis Blanton, Liesl Hagan, Stephanie J. Salyer

**Affiliations:** 1 Division of Global Health Protection, Center for Global Health, Centers for Disease Control and Prevention, 1600 Clifton Rd NE, Atlanta, GA 30329 USA; 2 National Center for Disease Control and Public Health, Tbilisi, Georgia; 3 Global Disease Detection – South Caucasus Regional Center, Centers for Disease Control and Prevention, Tbilisi, Georgia; 4 Division of Reproductive Health, National Center for Chronic Disease Prevention and Health Promotion, Centers for Disease Control and Prevention, 3005 Chamblee Tucker Rd, Atlanta, GA 30341 USA; 5 Division of Viral Hepatitis, National Center for HIV/AIDS, Viral Hepatitis, STD, and Tuberculosis Prevention, Centers for Disease Control and Prevention, 3 Corporate Blvd NE, Atlanta, GA 30329 USA

**Keywords:** Global Health, Non-communicable disease, Risk factors, Survey, Georgia, Global health security

## Abstract

**Background:**

Non-communicable diseases (NCDs), mainly cardiovascular diseases, are a substantial cause of mortality in the country of Georgia, accounting for approximately 93% of all deaths (standardized mortality rate 630.7 deaths per 100,000 persons per year) and an important threat to health security. We conducted a nationally representative survey examining the prevalence of NCDs and their risk factors as part of a 2015 Hepatitis C Virus (HCV) and Hepatitis B Virus (HBV) serosurvey.

**Methods:**

We conducted a cross-sectional serosurvey among adults aged ≥18 years using a stratified, multi-stage cluster design (*n* = 7000). We asked participants standardized questions from the Global Adult Tobacco Survey and the WHO STEPwise approach to Surveillance (STEPS) Survey. We also measured blood pressure and Body Mass Index for each participant. Weighted frequencies were computed for NCD and risk factor prevalence and compared to 2010 STEPS results.

**Results:**

Georgians reported high rates of smoking, alcohol use, elevated blood pressure, obesity, diabetes and cardiovascular disease. An estimated 27.1% (95% confidence interval [CI]: 25.3, 28.8%) of adults (51.5% of men and 6.0% of women) reported daily use of tobacco products and 27.5% (95% CI: 25.7, 29.2%) of adults (52.1% of men and 7.0% of women) reported binge drinking within the last 30 days. Physical measurements revealed that 37.5% (95% CI: 35.8, 39.3%) of adults had elevated blood pressure and 33.4% (95% CI: 31.8, 35.0%) had obesity. 5.4% (95% CI: 4.6, 6.2%) of adults had self-reported diagnosed diabetes and 15.3% (95% CI: 14.1, 16.6%) had self-reported diagnosed cardiovascular disease. From 2010 to 2015, the prevalence of obesity increased by 8.3 percentage points (95% CI: 5.9, 10.7%; *p* < 0.01) and the prevalence of elevated blood pressure increased by 4.1 percentage points (95% CI: 1.4, 6.8%; *p* < 0.01).

**Conclusions:**

Georgia has a high NCD burden, and results from the survey showed an increase in obesity and elevated blood pressure since 2010. The prevalence of other major NCDs have remained near levels reported in the 2010 STEPs survey. Comprehensive public health interventions are needed to control the heath security threats of major NCDs and their risk factors in the future.

**Electronic supplementary material:**

The online version of this article (10.1186/s12889-019-6785-2) contains supplementary material, which is available to authorized users.

## Background

Georgia is an Eastern European, middle-income country with 3.7 million residents [1]. Non-communicable diseases (NCDs) are the most substantial causes of mortality and morbidity in Georgia, accounting for an estimated 46,200 deaths in 2015 and resulting in an age-standardized mortality rate of 630.7 deaths per 100,000 persons per year [2]. That rate was 13th highest out of 50 countries in the World Health Organization’s (WHO) European Region, up from 16th in 2010, 25th in 2005, and 21st in 2000 [3]. A 2014 report revealed that NCDs accounted for approximately 93% of the country’s total mortality. In comparison, NCDs accounted for 90% of total mortality in the European region and 70% of total mortality worldwide [4]. Four main categories of NCDs, namely cardiovascular diseases, cancer, chronic respiratory diseases and diabetes, caused 88% of Georgia’s mortality, with 69% of mortality being caused by cardiovascular diseases alone.

A number of risk factors have been indicated in the high prevalence of NCDs, including excessive alcohol use, smoking, obesity, and elevated blood pressure, each of which has been shown to be associated with both NCDs and other negative health outcomes. Excessive alcohol use has been linked to high blood pressure, heart disease, stroke, liver disease, cancer, mental health problems, and alcohol dependence [5]. Smoking has been associated with an increased risk of coronary heart disease, stroke, type 2 diabetes mellitus, rheumatoid arthritis, and various cancers [6, 7]. A higher body mass index (BMI), beyond the normal weight range, is associated with increased morbidity and mortality from coronary heart disease, osteoarthritis, type 2 diabetes mellitus, hypertension, and certain types of cancer [8]. Hypertension is a major risk factor for cardiovascular disease and globally accounts for 54% of all strokes and 47% of all cases of ischemic heart disease [9].

The high mortality from NCDs and the strong association between NCDs and the identified risk factors has highlighted a need to measure national and subnational trends in NCD and NCD risk factor prevalence to inform prevention activities. The most recent NCD risk factor data assessing hypertension, obesity, smoking, and alcohol consumption in Georgia were collected from WHO STEPwise approach to surveillance (STEPS) assessments in 2010 and 2016. STEPs provides a standardized survey tool which includes a manual containing comprehensive guidelines for countries undertaking NCD risk factor surveys. The 2016 STEPs data indicated that 37.7% of the population had elevated blood pressure, with 55.4% of those being untreated [10]. About 64.6% of adults were considered overweight (BMI > 25 kg/m^2^), with 33.2% being obese (BMI > 30 kg/m^2^) [10]. Among males, 35.3% reported heavy episodic drinking and 51.5% reported smoking tobacco products daily [10].

In light of the 2010 and 2016 STEPS reports, the Georgian government took several significant steps to decrease the morbidity, disability and mortality caused by NCDs. In particular, the Multi-sectoral Coordination Council of Prevention and Control of NCDs was established under the Minister of Labor, Health and Social Affairs in December 2015. The 2017–2020 Action Plan and the NCD strategy were endorsed in January 2017. A national cancer registry and a birth registry were established (2015, 2016). Additionally, a sentinel surveillance system measuring nutrition and micronutrient deficiency was implemented, several studies on tobacco were conducted (2014, 2014, 2015), state routine surveillance was improved, national cancer screening programs (breast, cervical, colorectal and prostate cancer) were created (2011), and guidelines and protocols for major NCDs were developed.

In an additional effort to monitor Georgia’s progress in combating NCDs, a nationally representative, cross-sectional survey examining several NCDs and their risk factors was included as part of the Georgia Hepatitis C Virus (HCV) and Hepatitis B (HBV) Serosurvey in 2015. The objective of the NCD component of the survey was to estimate the prevalence of major NCDs and the major risk factors of NCDs in adults aged 18 years and above in Georgia. The survey built upon previously conducted STEPs surveys and included additional components including geographic estimates of cardiovascular diseases, chronic respiratory diseases, and cancers. This manuscript reports the results of the 2015 survey, including a comparison to the 2010 STEPs survey.

## Methods

The National Center for Disease Control and Public Health (NCDC) implemented a stratified, multi-stage cluster survey designed to yield national and subnational prevalence estimates for HCV and HBV, as well as estimates for various NCDs and NCD risk factors [11]. The survey population included all eligible adults aged 18 years and above who were living in a household in Georgia. Temporary household guests, homeless persons, those who were currently incarcerated or institutionalized were not eligible for selection. We calculated a sample size of 7000 people to attain 1% precision in our HCV prevalence estimate, assuming an estimated 6.7% anti-HCV seroprevalence [12], a design effect of 2, and an anticipated 70% response rate.

To select participants, we divided the country into 16 mutually exclusive sampling strata consisting of six major cities and ten regions. We did not include the autonomous regions of Abkhazia and Samachablo (South Ossetia) due to political conflict in the area. We selected 280 clusters, each representing one census sector as defined by Georgia’s National Statistics Office (GeoStat). Within each cluster, we conducted a systematic sample of 25 households. We divided the total number of households in the cluster by 25 and used a random starting point to begin sampling. Within each household, we applied the Kish method to randomly select one adult for participation [13].

We asked participants standardized questions from the Global Adult Tobacco Survey [14] and the WHO STEPwise approach to Surveillance (STEPS) Survey (version 2.1) [15], which was the same version used in Georgia’s 2010 STEPs survey. For survey questions concerning other chronic conditions like cancer, cardiovascular disease, and chronic respiratory disease, we developed questions from standard National Health and Nutritional Examination Survey (NHANES) wording [16]. In the analysis below, we report on results for four common NCD risk factors (current daily smoking, heavy episodic drinking, elevated blood pressure, obesity) and four major categories NCDs (chronic respiratory disease, cancer, diabetes, and cardiovascular disease).

We collected data on blood pressure and anthropometric measurements to estimate the proportion of adults that had elevated blood pressure or had obesity. Both measurements were carried out using standard equipment and the recommended WHO STEPS protocol [15]. We calculated BMI by dividing each participant’s weight (in kilograms) by their squared height (in meters). If a participant had a BMI > 30 kg/m^2^, we classified them as having obesity [15]. We used the mean of the 2nd and 3rd of three blood pressure measurements to estimate blood pressure. If a participant’s systolic blood pressure was ≥140 mmHg or their diastolic blood pressure was ≥90 mmHg, we classified them as having elevated blood pressure [15]. Current daily smoking, heavy episodic drinking, chronic respiratory disease, cancer, diabetes, and cardiovascular disease were assessed via participants’ self-report. The specific wording for each question corresponding to a self-reported indicator is listed in Table 1.

**Table 1:** Criteria for inclusion in prevalence calculation

Indicator	Indicator type	Question or criteria
NCDs		
Current daily smoking	self-report	Do you currently smoke tobacco on a daily basis, less than daily, or not at all?
Heavy episodic drinking	self-report	(For men) During the past 30 days, did you had five or more standard alcoholic drinks in a single occasion?
		(For women) During the past 30 days, did you had four or more standard alcoholic drinks in a single occasion?
Elevated Blood Pressure	physical measurement	systolic blood pressure ≥ 140 mmHg or diastolic blood pressure ≥ 90 mmHg
Obesity	physical measurement	BMI > 30 kg/m^2^
NCD risk factors		
Cardiovascular disease	self-report	Have you ever been told by a doctor or other health worker that you have cardiovascular disease?
Cancer	self-report	Have you ever been told by a doctor or other health worker that you have cancer?
Chronic respiratory disease	self-report	Have you ever been told by a doctor or other health worker that you have asthma or lung disease or COPD?
Diabetes	self-report	Have you ever been told by a doctor or other health worker that you have diabetes?

We conducted statistical analysis in SAS (version 9.4, Cary, NC) and accounted for the probability of selection at the cluster, household, and individual levels using survey weights. The weights were calibrated to represent Georgia’s national population in terms of sex, age, and geographic distribution based on 2014 census data. We computed descriptive statistics, including weighted prevalence estimates with 95% confidence intervals (CI), to describe the outcomes of interest. Two sample z-tests for proportions (α = 0.05) were used to quantify the differences in prevalence over time. Choropleth maps describing the geographic spread of NCDs and NCD risk factors were created in QGIS 2.18.10.

This activity was reviewed in accordance with CDC human subjects review procedures and was determined to be non-research, public health surveillance.

## Results

Of the 7000 adults selected for the survey, 6296 (89.9%) consented to participate, with response rates exceeding 70% in all 16 strata. The adults ranged from 18 to 102 years old and the median age was 45. 53.8% were female, and 56.7% lived in urban areas (Table 2). Most (90.8%) had completed education through secondary school or higher, and 19.5% were unemployed at the time of the survey. Approximately 60.8% reported an annual household income less than 12,000 Georgian Lari ($6797 USD).

**Table 2:** Demographic characteristics among Georgian adults, Georgia NCD survey, 2015

Demographic Characteristics	Unweighted sample size	Population-weighted percentage
	n	% (95% CI)
Overall		
Age (years)		
18–29	1115	19.4 (18.1, 20.7)
30–44	1725	29.0 (27.3, 30.7)
45–59	1662	25.5 (24.0, 27.0)
60+	1790	26.1 (24.5, 27.7)
Missing	4	0.05 (< 0.01, 0.1)
Sex		
Males	3868	53.8 (52.0, 55.5)
Females	2428	46.2 (44.5, 48.0)
Missing	0	0
Residency		
Urban	3350	56.7 (52.7, 60.6)
Rural	2946	43.3 (39.4, 47.3)
Missing	0	0
Education		
Completed less than elementary/primary school	43	0.7 (0.5, 1.1)
Completed elementary/primary school	612	8.5 (7.3, 9.8)
Completed secondary school	2567	40.2 (38.1, 42.3)
Completed professional/technical school	1157	16.6 (15.3, 18.0)
Completed university/college or higher	1912	34.0 (31.6, 36.4)
Missing	5	0.09 (< 0.01, 0.2)
Employment Status		
Employed	2120	37.8 (35.6, 39.9)
Student	172	3.6 (2.9, 4.4)
Homemaker	1483	19.1 (17.7, 20.6)
Retired	1405	20.0 (18.7, 21.5)
Unemployed	1110	19.5 (18.0, 21.1)
Missing	6	0.08 (0.02, 0.14)
Household income		
≤ 6000 GEL/year (≤ 4400 USD)	2960	43.7 (41.2, 46.2)
6001–12,000 GEL/year (4400–6800 USD)	953	17.1 (15.5, 18.7)
12,001–24,000 GEL/year (6800–13,600 USD)	724	11.6 (10.4, 12.9)
> 24,000 GEL/year (> 13,600 USD)	1339	21.6 (19.4, 23.9)
Missing	320	5.9 (4.8, 7.0)

The majority of the individuals surveyed were vulnerable to morbidity from NCDs, with 72.3% (95% CI: 70.7, 73.8%) of adults reporting at least one of four NCD risk factors. Heightened risk existed among the 39.3% (95% CI: 37.3, 41.3%) of people reporting at least two NCD risk factors, and the 12.2% (95% CI: 11.0, 13.4%) of people reporting at least three NCD risk factors (Table 3). The most prevalent risk factor was elevated blood pressure, which was estimated to impact 37.5% (95% CI: 35.8, 39.3%) of the population according to physical measurements conducted during the survey (Table 2). Additionally, an estimated 33.4% (95% CI: 31.8, 35.0%) of adults had obesity, 27.5% (95% CI: 25.7, 29.2%) reported heavy episodic drinking in the last 30 days, and 27.1% (95% CI: 25.3, 28.8%) reported currently smoking tobacco products on a daily basis.

**Table 3:** Overall prevalence of NCDs and NCD risk factors

	n	% (95% CI)
NCD risk factors		
Obesity*	2103	33.4 (31.8, 35.0)
Current daily smoking	1707	27.1 (25.3, 28.8)
Heavy episodic drinking†	1731	27.5 (25.7, 29.2)
Elevated Blood Pressure‡	2361	37.5 (35.8, 39.3)
At least one of the above risk factors	4230	72.3 (70.7, 73.8)
At least two of the above risk factors	2188	39.3 (37.3, 41.3)
At least three of the above risk factors	613	12.2 (11.0, 13.4)
At least four of the above risk factors	114	2.3 (1.7, 3.0)
NCDs		
Cardiovascular Disease	964	15.3 (14.1, 16.6)
Cancer	57	0.9 (0.6, 1.2)
Chronic Respiratory Disease	246	3.9 (3.2, 4.7)
Diabetes	340	5.4 (4.6, 6.2)
At least one of the above NCDs	1465	22.1 (20.5, 23.6)
At least two of the above NCDs	207	3.0 (2.4, 3.5)
At least three of the above NCDs	17	0.4 (0.1, 0.7)
At least four of the above NCDs	0	0

*Obesity is defined as BMI ≥30 kg/m^2^

†For men, heavy episodic drinking is defined as consuming 5 or more standard alcoholic drinks in a single occasion in the last 30 days

†For women, a heavy episodic drinking is defined as consuming 4 or more standard alcoholic drinks in a single occasion in the last 30 days

‡Elevated blood pressure is systolic blood pressure ≥ 140 mmHg or diastolic blood pressure ≥ 90 mmHg

A high prevalence of NCDs was also observed in this survey. An estimated 22.1% of adults reported at least one of the four main categories of NCDs (cardiovascular diseases, cancers, chronic respiratory diseases, and diabetes), 3.0% of reported at least two and 0.4% reported at least three. The most prevalent NCD was cardiovascular disease, which was reported among 15.3% (95% CI: 14.1, 16.6%) of the population. In decreasing order of prevalence, respondents also reported diabetes, 5.4% (95% CI: 4.6, 6.2%), chronic respiratory diseases, 3.9% (95% CI: 3.2, 4.7%), cancer, 0.9% (95% CI: 0.6, 1.2%).

A comparison between our 2015 dataset and the 2010 STEPS survey data revealed mixed results in Georgia’s effort to reduce the prevalence of NCD risk factors (Table 4). Over the five year period between surveys, the prevalence of obesity increased by 8.3 percentage points (95% CI: 5.9, 10.7%; z = 6.8, *p* < 0.01). The prevalence of elevated blood pressure for females increased by 3.2 percentage points (95% CI: 0.4, 6.0%; z = 2.2, *p* < 0.05). The prevalence of heavy episodic drinking among males increased by 2.3 percentage points (95% CI: -2.8, 7.4%; z = 0.9, *p* = 0.37), although the apparent increase was not statistically significant and may have been due to sampling error. Among females, reported heavy episodic drinking actually decreased by 3.3 percentage points (95% CI: 1.1, 5.5%; z = − 2.9, p < 0.01). Current daily tobacco use also decreased by 0.6% (95% CI: -2.0, 3.2%; z = 0.2, *p* = 0.64), although again, the apparent decrease was not significant.

**Table 4:** Change in non-communicable disease risk factor prevalence, 2010–2015

Disease	2010 STEPS survey	2015 HCV survey	Change	z	*p*-value
	% (95% CI)	% (95% CI)	% (95% CI)		
Obesity*	25.1 (23.3, 26.8)	33.4 (31.8, 35.0)	8.3 (5.9, 10.7)	6.84	< 0.01
Male	21.8 (19.3, 24.3)	29.0 (26.6, 31.4)	7.2 (3.7, 10.7)	4.06	< 0.01
Female	28.5 (26.6, 30.3)	37.1 (35.1, 39.2)	8.6 (5.8, 11.4)	6.12	< 0.01
Current daily smoking	27.7 (25.8, 29.5)	27.1 (25.3, 28.8)	−0.6 (−3.2, 2.0)	0.46	0.64
Male	51.1 (48.1, 54.0)	51.5 (48.5, 54.6)	0.4 (−3.8, 4.6)	0.17	0.85
Female	4.0 (2.9, 5.0)	6.0 (4.7, 7.3)	2.0 (0.3, 3.7)	2.36	0.02
Heavy episodic drinking†	NR	27.5 (25.7, 29.2)	NA	NA	NA
Male	49.8 (45.7, 53.9)	52.1 (49.2, 55.0)	2.3 (−2.8, 7.4)	0.89	0.37
Female	10.3 (8.5, 12.0)	7.0 (5.7, 8.3)	−3.3 (−5.5, −1.1)	2.94	< 0.01
Elevated Blood Pressure (measured)‡	33.4 (31.4, 35.5)	37.5 (35.8, 39.3)	4.1 (1.4, 6.8)	2.97	< 0.01
Male	37.1 (34.0, 40.3)	42.7 (39.9, 45.5)	5.6 (1.4, 9.8)	2.59	< 0.01
Female	29.8 (27.9, 31.8)	33.0 (31.0, 35.1)	3.2 (0.4, 6.0)	2.21	0.03

*Obesity is defined as BMI ≥30 kg/m^2^

†For men, heavy episodic drinking is defined as consuming 5 or more standard alcoholic drinks in a single occasion in the last 30 days

†For women, a heavy episodic drinking is defined as consuming 4 or more standard alcoholic drinks in a single occasion in the last 30 days

‡Elevated blood pressure is systolic blood pressure ≥ 140 mmHg or diastolic blood pressure ≥ 90 mmHg

**Fig. 1:**
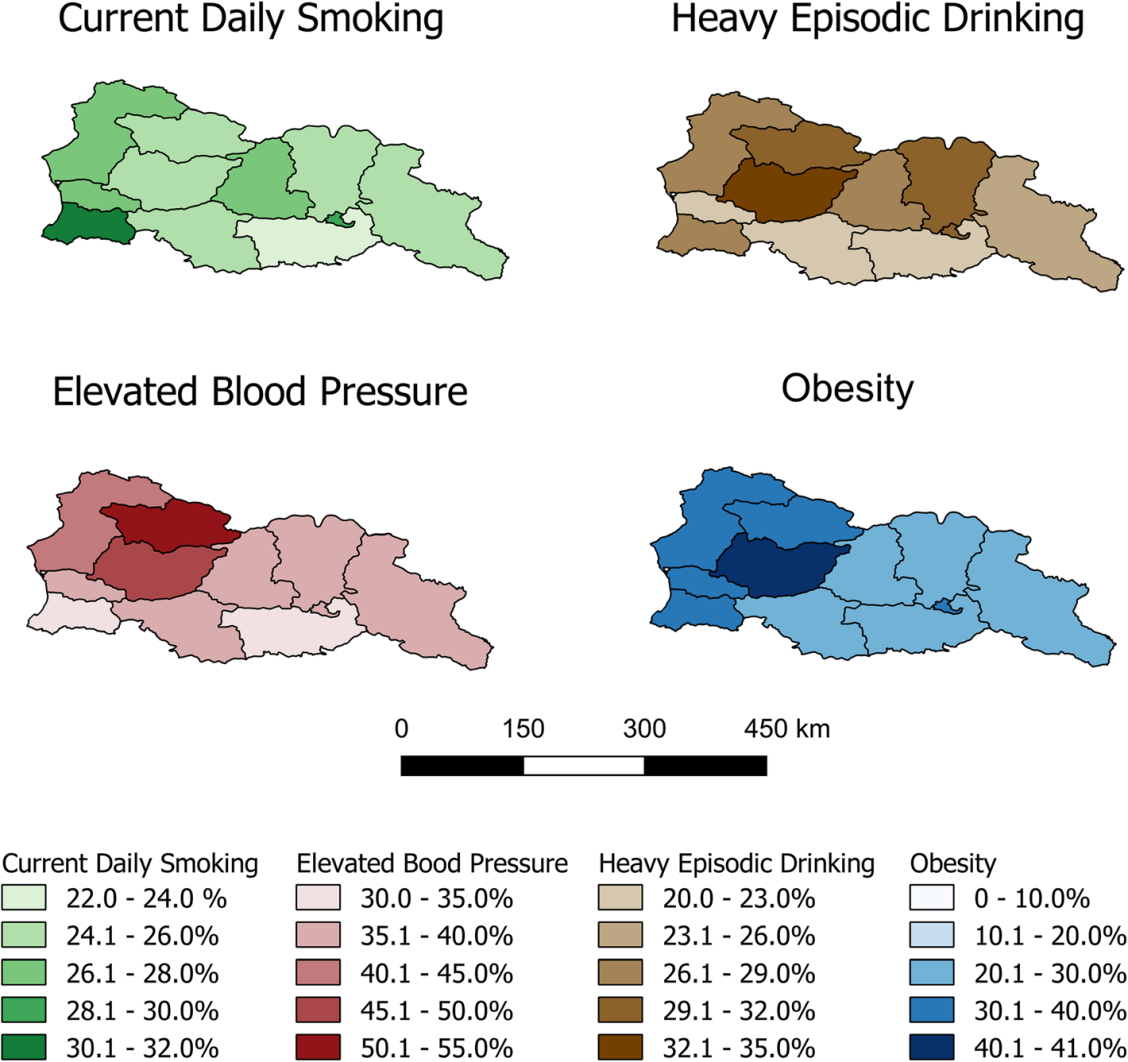
Prevalence of non-communicable disease risk factors by region. The four choropleth maps show the prevalence of current daily smoking (green), heavy episodic drinking in the last 30 days (brown), elevated blood pressure (red), and obesity (blue) by region in Georgia. In each map, the darker colors represent a higher prevalence of disease

**Fig. 2:**
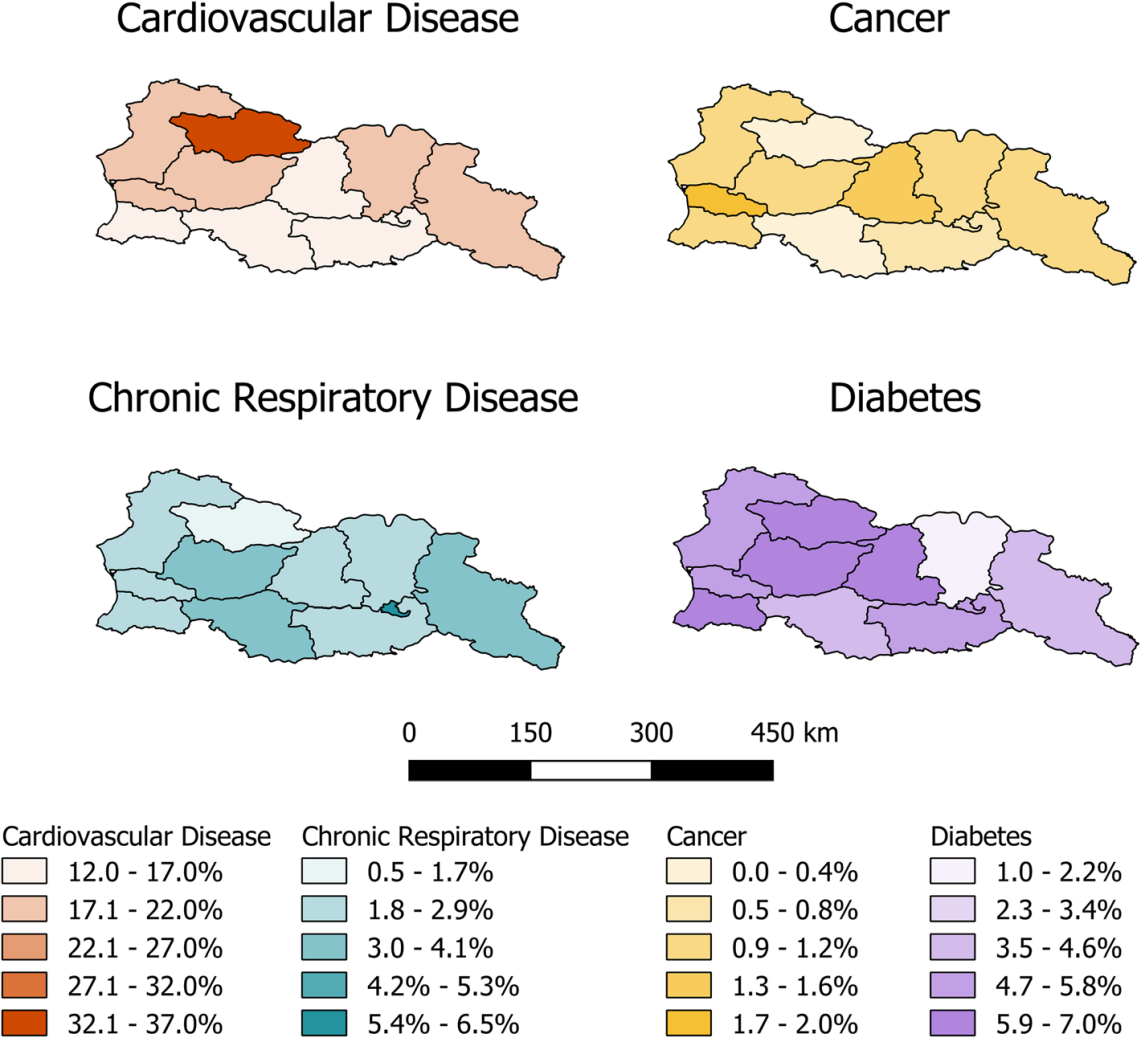
Prevalence of non-communicable diseases by district. The four choropleth maps show the prevalence of cardiovascular disease (orange), cancer (gold), chronic respiratory disease (aqua) and diabetes (purple) by region in Georgia. In each map, the darker colors represent a higher prevalence of disease

An analysis of the geographical variation in NCDs and NCD risk factors (Figs. 1 and 2) found that the region of Imereti had the highest prevalence of obesity (40.1, 95% CI: 35.9, 44.2%), the highest reported prevalence of heavy episodic drinking (32.1, 95% CI: 28.3, 35.8%), and the second highest prevalence of elevated blood pressure (45.5, 95% CI: 42.3, 48.6%). The only region with higher elevated blood pressure (50.9, 95% CI: 44.8, 57.1%) was Racha-Lechkumi, which also reported the highest prevalence of cardiovascular disease (36.2, 95% CI: 30.4, 42.0%) and diabetes (6.7, 95% CI: < 0.1, 15.2%). The proportion of people who reported daily tobacco use was highest in Tbilisi (30.0, 95% CI: 25.7, 34.2%) and Ajaria (30.5, 95% CI: 26.9, 34.1%), the regions containing the first second largest cities in Georgia, respectively. Tbilisi also reported the highest prevalence of chronic respiratory disease (6.4, 95% CI: 4.3, 8.4%), perhaps partially because of the aforementioned high smoking rate. The highest reported prevalence of cancer occurred in the region of Guria (1.9, 95% CI: < 0.1, 4.3%). A full analysis of geographical variation is provided (Additional file 1: Table S1).

Wide disparities in the prevalence of NCD and NCD risk factors between gender and age groups were also apparent. Men engaged in much higher rates of unhealthy behaviors, most notably smoking and heavy episodic drinking. 51.5% (95% CI: 48.5, 54.6%) of men reported smoking tobacco products daily, compared to 6.0% (95% CI: 4.7, 7.3%) of females. 52.1% (95% CI: 49.2, 55.0%) of men reported heavy episodic drinking in the last 30 days compared to 7.0% (5.7, 8.3%) of women. Women were more likely to have obesity, with 37.1% (95% CI: 35.1, 39.2%) of women having a BMI > 30 kg/m^2^, compared to 29.0% (95% CI: 26.6, 31.4%) of men. Compared to those under 45 years of age, older adults (45+) were more likely to have elevated blood pressure and obesity, and were more likely to report cardiovascular disease, cancer, chronic respiratory disease, and diabetes. A full analysis of NCD and NCD risk factor prevalence by age group and gender is provided (Additional file 2: Table S2).

## Discussion

The results of this nationally representative survey highlight the high burden of common NCDs and their major risk factors in Georgian adults. Compared to many of its European peers, Georgia has not yet been successful in limiting tobacco use, obesity, diabetes, or cardiovascular disease. While significant steps have been made to improve NCD surveillance and care in Georgia, measurable decreases in risk factors and disease have not yet been observed. Modest victories, including a decrease in daily tobacco use and heavy episodic drinking (among women) since 2010, provide a reason for optimism and offer a blueprint for future action. Setbacks, including the increase in obesity and hypertension, reiterate the importance of prevention efforts and underscore the need to bolster existing interventions.

While the overall, nationwide burden of NCDs is high, it is not equally distributed across the population. Regional and demographic differences in NCD prevalence underlie differences in lifestyle, socioeconomic status, and access to healthcare. Older residents tend to report diminished outcomes compared to their younger counterparts. Wide gender disparities were evident, with men reporting higher levels of tobacco use and heavy episodic drinking, but women reporting higher prevalence of cardiovascular diseases and cancers. With such differences in mind, a concerted effort to focus potential health interventions on specific high-risk populations may be warranted. Targeted interventions can provide a dual benefit; they are likely to be more cost efficient while also serving to promote health equity.

Georgia’s increasing obesity prevalence is consistent with global and regional trends, and could be attributed to a number of potential factors, including an aging population and continued high rates of alcohol consumption [17]. Adults who were 45 years or older, especially women, had a much higher obesity prevalence than those in the younger age groups. Interventions aimed at improving physical activity and healthy dietary intake, particularly those focused among high-risk demographic groups, may help to ease the obesity burden in Georgia.

The observed increase in elevated blood pressure was also notable, and may have been even greater than estimated. In the 2010 STEPS survey, an individual was labeled hypertensive if their measured blood pressure was elevated (≥140 SBP or ≥ 90 DBP) or if they were on blood pressure medications. This study did not collect information on blood pressure medication usage so it did not include those with normal pressure (< 140 SBP and < 90 DBP) who were taking blood pressure medications as the 2010 STEPS rates did. This difference in methodology would underestimate the rate of hypertension found in 2015 compared with 2010, assuming persons on anti-hypertensive therapy would have good blood pressure control. Efforts to control hypertension with medical care and education regarding nutrition, staying active, and moderating alcohol use may help reduce hypertension [18] in the population.

There were several limitations of this study. First, we used self-reported data to estimate the prevalence of NCDs, which could potentially be subject to inaccuracies due to recall bias. Second, social desirability bias may have occurred due to the potential stigmatization of certain behaviors and conditions, including questions about behaviors such as smoking and alcohol consumption. Third, we collected data on broadly defined disease categories, such as diabetes, cancer, and cardiovascular disease. Thus, we are unable to make inferences on specific subtypes of those diseases, for example comparisons between type 1 and type 2 diabetes, or comparisons between heart disease and stroke. Fourth, while we used sampling weights designed to adjust for non-response, the moderate response rate may have still resulted in some degree of non-response bias. Finally, the estimated prevalence of hypertension was not directly comparable to the 2010 STEPS survey, due to the methodological differences described above. Despite these limitations, this study adds updated information on the NCD burden and identifies the trends of major NCD risk factors in Georgian adults. The NCD risk factor prevalence estimates generated by the 2015 survey have since been corroborated by similar 2016 STEPs survey estimates (when direct comparisons were possible).

## Conclusions

The NCD risk factors listed in this study are each associated with multiple NCDs. Thus, improving the prevention of a single risk factor could result in a decreased prevalence of multiple NCDs. Conversely, an increase in a single risk factor could lead to multiple negative health outcomes. Continued investment in comprehensive prevention and control interventions could be considered to combat these negative outcomes. A concerted national effort to enact prudent, evidence-based interventions could improve quality of life, reduce mortality, strengthen global health security, and counteract the economic costs associated with the high burden of NCDs.

## Additional files

Additional file 1: **Table S1.** NCD and NCD risk factor prevalence by region (DOCX 15 kb)

Additional file 2: **Table S2**. NCD and NCD risk factor prevalence by gender and age group (DOCX 16 kb)

## Abbreviations

BMI: Body mass index
CDC: Centers for Disease Control and Prevention
CI: Confidence interval
DBP: Diastolic blood pressure
GeoStat: Georgia’s National Statistics Office
HBV: Hepatitis B Virus
HCV: Hepatitis C Virus
HIV/AIDS: Human immunodeficiency virus infection and acquired immune deficiency syndrome
NCD: Non-communicable disease
NCDC: National Center for Disease Control and Public Health
NHANES: National Health and Nutritional Examination Survey
QGIS: Quantum Geographic Information System
SAS: Statistical analysis system
STD: Sexually transmitted disease
STEPS: STEPwise approach to surveillance
WHO: World Health Organization
