# Supplementary material for: The burden of non-communicable diseases and their related risk factors in the country of Georgia, 2015
Source: BMC Public Health. 2019 May 10;19(Suppl 3):479. doi: 10.1186/s12889-019-6785-2 (PMC6696664; doi:10.1186/s12889-019-6785-2)
Supplement: Supplementary file 1 — Table S1. NCD and NCD risk factor prevalence by region (DOCX 15 kb) [file 12889_2019_6785_MOESM1_ESM.docx]

| **Table S1. NCD and NCD risk factor prevalence by region** | | | | | | | | | |
| --- | --- | --- | --- | --- | --- | --- | --- | --- | --- |
| **Region** |  | **Cardiovascular Disease** | **Cancer** | **Chronic Respiratory Disease** | **Diabetes** | **Obesity*** | **Current daily smoking** | **Heavy episodic drinking†** | **Elevated Blood Pressure**‡ |
|  | n | % (95% CI) | % (95% CI) | % (95% CI) | % (95% CI) | % (95% CI) | % (95% CI) | % (95% CI) | % (95% CI) |
| Adjara | 684 | 13.9 (10.3, 17.5) | 1.0 (0.3, 1.7) | 2.9 (1.3, 4.5) | 5.9 (3.8, 8.0) | 34.4 (29.2, 39.7) | 30.5 (26.9, 34.1) | 27.2 (22.7, 31.7) | 34.2 (29.3, 39.1) |
| Guria | 209 | 19.4 (12.3, 26.6) | 1.9 (<0.1, 4.3) | 2.9 (<0.1, 6.0) | 4.8 (1.3, 8.4) | 37.1 (27.6, 46.6) | 27.7 (16.8, 38.6) | 22.6 (16.5, 28.7) | 35.8 (28.9, 42.7) |
| Imereti | 1089 | 18.4 (15.1, 21.7) | 1.0 (0.4, 1.6) | 3.6 (2.2, 4.9) | 6.5 (4.4, 8.6) | 40.1 (35.9, 44.2) | 24.9 (21.2, 28.6) | 32.1 (28.3, 35.8) | 45.5 (42.3, 48.6) |
| Kakheti | 889 | 19.1 (15.4, 22.7) | 1.2 (0.5, 2.0) | 3.3 (1.8, 4.7) | 4.4 (2.6, 6.3) | 29.5 (26.0, 33.0) | 24.3 (19.9, 28.8) | 24.3 (19.2, 29.4) | 35.1 (29.9, 40.4) |
| Kvemo Kartli | 831 | 12.7 (9.8, 15.6) | 0.6 (<0.1, 1.3) | 1.9 (1.0, 2.8) | 5.5 (3.3, 7.6) | 28.3 (24.5, 32.1) | 23.6 (19.7, 27.6) | 20.0 (15.8, 24.1) | 31.7 (27.7, 35.7) |
| Mtskheta-Mtianeti | 161 | 19.6 (11.4, 27.7) | 0.9 (<0.1, 1.9) | 2.6 (0.2, 5.0) | 1.5 (<0.1, 3.6) | 29.3 (17.3, 41.3) | 25.3 (14.1, 36.5) | 30.8 (21.2, 40.4) | 38.4 (25.8, 51.0) |
| Racha-Lechkhumi and Kvemo Svaneti | 67 | 36.2 (30.4, 42.0) | 0 (NA) | 0.5 (<0.1, 1.6) | 6.7 (<0.1, 15.2) | 35.8 (22.8, 48.9) | 24.3 (15.6, 33.0) | 30.1 (16.2, 44.1) | 50.9 (44.8, 57.1) |
| Samegrelo-Zemo Svaneti | 826 | 20.3 (16.5, 24.0) | 1.0 (0.1, 1.9) | 2.3 (1.0, 3.5) | 5.2 (3.1, 7.3) | 34.7 (30.9, 38.5) | 26.5 (22.5, 30.4) | 27.9 (24.6, 31.3) | 42.1 (38.0, 46.1) |
| Samtskhe-Javakheti | 274 | 12.2 (8.0, 16.5) | 0.2 (<0.1, 0.7) | 4.0 (0.4, 7.6) | 3.7 (0.8, 6.6) | 28.8 (22.0, 35.7) | 24.4 (13.8, 35.0) | 20.5 (12.9, 28.1) | 39.6 (31.0, 48.3) |
| Shida Kartli | 416 | 15.2 (10.4, 20.0) | 1.4 (0.2, 2.5) | 2.4 (0.4, 4.4) | 6.1 (3.7, 8.5) | 28.8 (23.4, 34.1) | 26.2 (21.8, 30.6) | 26.1 (19.4, 32.8) | 37.9 (30.4, 45.3) |
| Tbilisi | 744 | 12.0 (9.3, 14.7) | 0.7 (0.1, 1.4) | 6.4 (4.3, 8.4) | 5.4 (3.4, 7.4) | 33.9 (30.4, 37.4) | 30.0 (25.7, 34.2) | 30.3 (26.0, 34.5) | 35.4 (31.3, 39.5) |

*Obesity is defined as BMI ≥30 kg/m^2^

**†**For men, heavy episodic drinking is defined as consuming 5 or more standard alcoholic drinks in a single occasion in the last 30 days.

**†**For women, a heavy episodic drinking is defined as consuming 4 or more standard alcoholic drinks in a single occasion in the last 30 days

‡Elevated blood pressure is systolic blood pressure ≥140 mmHg or diastolic blood pressure ≥90 mmHg
