# Supplementary material for: The burden of non-communicable diseases and their related risk factors in the country of Georgia, 2015
Source: BMC Public Health. 2019 May 10;19(Suppl 3):479. doi: 10.1186/s12889-019-6785-2 (PMC6696664; doi:10.1186/s12889-019-6785-2)
Supplement: Supplementary file 2 — Table S2. NCD and NCD risk factor prevalence by gender and age group (DOCX 16 kb) [file 12889_2019_6785_MOESM2_ESM.docx]

| **Table S2. NCD and NCD risk factor prevalence by gender and age group** | | | | | | | | | | | |
| --- | --- | --- | --- | --- | --- | --- | --- | --- | --- | --- | --- |
|  |  |  | **Cardiovascular Disease** | **Cancer** | **Chronic Respiratory Disease** | **Diabetes** | **Obesity*** | **Current daily smoking** | **Heavy episodic drinking†** | **Elevated Blood Pressure**‡ |  |
|  |  | n | mean (95% CI) | mean (95% CI) | mean (95% CI) | mean (95% CI) | mean (95% CI) | mean (95% CI) | mean (95% CI) | mean (95% CI) |  |
| **Overall** |  |  |  |  |  |  |  |  |  |  |  |
| *Age* | All | 6287 | 15.3 (14.1, 16.6) | 0.9 (0.6, 1.2) | 3.9 (3.2, 4.7) | 5.4 (4.6, 6.2) | 33.4 (31.8, 35.0) | 27.1 (25.3, 28.8) | 27.5 (25.7, 29.2) | 37.5 (35.8, 39.3) |  |
| (years) | 18-29 | 1115 | 0.9 (0.1, 1.7) | 0.2 (<0.1, 0.6) | 1.3 (0.5, 2.1) | 0.8 (<0.1, 1.7) | 14.6 (11.7, 17.6) | 32.3 (28.0, 36.6) | 34.7 (30.8, 38.5) | 13.7 (10.7, 16.7) |  |
|  | 30-44 | 1723 | 3.7 (2.6, 4.8) | 0.3 (<0.1, 0.8) | 3.1 (1.8, 4.4) | 2.1 (1.2, 3.0) | 27.4 (24.6, 30.2) | 35.7 (32.5, 39.0) | 34.8 (31.3, 38.2) | 24.1 (21.1, 27.1) |  |
|  | 45-59 | 1660 | 15.3 (13.2, 17.4) | 1.1 (0.5, 1.7) | 4.4 (2.8, 6.1) | 6.7 (5.0, 8.4) | 44.9 (41.4, 48.4) | 29.0 (25.6, 32.4) | 28.7 (25.5, 31.9) | 46.1 (42.6, 49.6) |  |
|  | 60+ | 1789 | 39.1 (36.1, 42.1) | 1.9 (1.1, 2.7) | 6.2 (4.5, 8.0) | 11.1 (9.0, 13.2) | 42.8 (39.5, 46.2) | 11.7 (9.6, 13.8) | 13.2 (11.0, 15.3) | 61.7 (58.1, 65.3) |  |
| **Male** |  |  |  |  |  |  |  |  |  |  |  |
| *Age* | All | 2425 | 12.2 (10.6, 13.9) | 0.3 (0.1, 0.5) | 4.4 (3.1, 5.6) | 5.8 (4.6, 7.0) | 29.0 (26.6, 31.4) | 51.5 (48.5, 54.6) | 52.1 (49.2, 55.0) | 42.7 (39.9, 45.5) |  |
| (years) | 18-29 | 473 | 0.8 (<0.1, 2.1) | 0 (NA) | 1.2 (0.3, 2.1) | 1.5 (0.0, 3.3) | 18.0 (13.5, 22.6) | 56.3 (48.9, 63.7) | 56.8 (50.6, 63.0) | 21.5 (16.2, 26.7) |  |
|  | 30-44 | 665 | 3.3 (1.8, 4.9) | 0.2 (<0.1, 0.4) | 4.3 (2.3, 6.3) | 3.2 (1.6, 4.9) | 28.7 (24.3, 33.2) | 64.6 (59.5, 69.6) | 62.0 (57.1, 66.9) | 35.7 (30.9, 40.5) |  |
|  | 45-59 | 660 | 15.1 (11.6, 18.5) | 0.2 (<0.1, 0.7) | 5.6 (2.7, 8.5) | 8.2 (5.2, 11.1) | 37.4 (32.0, 42.8) | 53.4 (47.7, 59.2) | 54.9 (49.4, 60.4) | 53.9 (48.8, 58.9) |  |
|  | 60+ | 627 | 33.5 (29.3, 37.7) | 0.8 (0.3, 1.4) | 6.3 (3.4, 9.1) | 10.6 (7.8, 13.4) | 30.2 (25.9, 34.5) | 26.6 (22.2, 31.0) | 31.1 (26.4, 35.7) | 60.2 (54.8, 65.5) |  |
| **Female** |  |  |  |  |  |  |  |  |  |  |  |
| *Age* | All | 3862 | 17.9 (16.1, 19.7) | 1.4 (0.9, 1.9) | 3.5 (2.6, 4.4) | 5.0 (4.0, 6.1) | 37.1 (35.1, 39.2) | 6.0 (4.7, 7.3) | 7.0 (5.7, 8.3) | 33.0 (31.0, 35.1) |  |
| (years) | 18-29 | 642 | 1.0 (0.1, 1.8) | 0.4 (<0.1, 1.2) | 1.3 (0.2, 2.5) | 0 (NA) | 11.0 (8.2, 13.9) | 6.1 (3.7, 8.5) | 11.5 (8.2, 14.9) | 5.3 (2.7, 7.9) |  |
|  | 30-44 | 1058 | 4.0 (2.5, 5.5) | 0.5 (<0.1, 1.0) | 2.1 (0.5, 3.6) | 1.1 (0.3, 1.9) | 26.2 (22.6, 29.7) | 9.0 (6.1, 12.0) | 10.4 (7.0, 13.8) | 13.4 (10.5, 16.3) |  |
|  | 45-59 | 1000 | 15.5 (12.7, 18.3) | 1.8 (0.8, 2.8) | 3.4 (2.0, 4.8) | 5.4 (3.8, 7.0) | 51.7 (47.6, 55.7) | 7.0 (4.5, 9.6) | 6.2 (3.9, 8.5) | 39.1 (34.7, 43.5) |  |
|  | 60+ | 1162 | 42.7 (38.7, 46.7) | 2.6 (1.4, 3.8) | 6.2 (4.1, 8.3) | 11.5 (8.6, 14.3) | 51.2 (46.9, 55.4) | 2.2 (0.7, 3.7) | 1.8 (0.5, 3.1) | 62.6 (58.3, 66.9) |  |

*Obesity is defined as BMI ≥30 kg/m^2^

**†**For men, heavy episodic drinking is defined as consuming 5 or more standard alcoholic drinks in a single occasion in the last 30 days.

**†**For women, a heavy episodic drinking is defined as consuming 4 or more standard alcoholic drinks in a single occasion in the last 30 days

‡Elevated blood pressure is systolic blood pressure ≥140 mmHg or diastolic blood pressure ≥90 mmHg
